# Minor Mental Maladies

**Published:** 1863-08

**Authors:** Andrew McFarland


### CHICAGO MEDICAL EXAMINER.

N. S. DAVIS, M.D., EDITOR.

VOL. IV. AUGUST, 1863. NO. 8.

### Original Contributions.

### ARTICLE XIX.

### MINOR MENTAL MALADIES.

BY ANDREW McFARLAND, M.D.

Read before the Illinois State Medical Society, May 6, and the American Association of Superintendents of American Institutions for the Insane, May 19, 1863.

It is a constantly recurring question, how far the terms *healthy* and *sane* should be regarded as the expressions of a strictly positive idea.

The solecism that a person is partially sick, is seldom heard; but the term, partially insane, is so common as to pass without a question. The boundary between physical health and disease is supposed to be well defined. The determinate point at which a person shall be called physically *sick* has a certain precision about it, it being a point which the person affected can define for himself. All there is abitrary about it, arises from peculiarities of mental constitution, which may lead one individual to fix the period at an earlier date than that chosen by another. In all mental affections, however, the opinions of those who pass judgment upon themselves are regarded more in the light of *ex parte* proceedings, requiring confirmation by the judgment of others.

The "thin partitions," recognized in Dryden's couplet, fade away almost entirely in regard to a great number of cases of mental impairment, creating a border territory, on the domain of which reason or madness may be supposed to hold sway, much according to the caprice in opinion of whoever may, for the time being, pass judgment thereupon.

Perpetual embarrassments and social disturbances are taking place from the sayings and doings of individuals, who, while occupying the wrong side of the border line, claim the prerogatives exclusively appertaining to the occupants of the other, neither themselves nor those affected by their acts perceiving the wrong stand-point from which such acts have their issue. Probably, physicians, more frequently than any other class, are subject to such embarrassments, and it will be the present object to pass in review some of those abnormal mental states which are apt to impose upon the practitioner, and sometimes, I apprehend, seriously warp his judgment.

It is presumed that, in the all-important matter of health, the statement of the patient to his physician will be as strictly truthful as language can make it. The physician always having this in mind, becomes, at the bedside of his patient, the most credulous of men, usually accepting, without question, all statements of facts on matters not otherwise capable of demonstration. It is only where the statement is most strikingly at variance with probability that his suspicions are ever aroused, and then even so tardily and feebly that he may be the victim of most egregious impositions for a long period. In his department, as in all others, the most successful imposter is the one who is himself deceived, and the earnestness, profound sincerity, and ceaseless importunity with which some self-deception may be forced on the unsuspecting physician, will command for a palpable error all the attention due to matters of highest earthly consequence.

Sources of error more trifling than any which proceed from a patient's imagination, may warp the judgment of the unthinking physician. Even the temperament of the messenger, whose only office is to convey the summons of the sick man to the doctor, may be potent enough to sway the latter into a prejudgment of the case in matters of diagnosis and treatment. How many physicians are there who could be aroused from deep slumber and hurried to the bed of a sick man by some such Mercurius of a messenger, as we have seen, without being prepared with a prognosis, diagnosis, and treatment, hard to be abandoned, which had been colored, at least, by the glowing imagination of such an insignificant functionary?

The mere idea of being diseased, as it impresses the mind of one to whom health is the natural state, is productive of an abnormal mental condition. The stays and supports which the mind receives in the conception of a variety of ideas, are missed when one engrossing idea occupies the attention. The estimate which the physician has gathered of his patient while in a state of health, fails him when the individual assumes the relation of a patient. Timidity, want of confidence, and even incivility are found to have taken the place of the manliness in which the same individual was before supposed to abound.

There are circumstances attending the mere occupancy of a sick bed, calculated, irrespective of the form of the disease, to place one in a factitious state of mind, which the physician will do well to consider. Even the disrobing and going to bed puts a person in an unnatural relation to the erect and active world about him. A man can not even exchange his roundabout and boots for a dressing-gown and slippers without being made, in feeling, at least, somewhat effeminate by the act, and what an abatement in his manliness is there when he is reduced,—a single garment only excepted,—to the original suit in which he made his mundane début. It was regarded as one of the clearest proofs of the regal dignity of Louis XIV. that he could dress and undress, take his physic and endure its consequences, while surrounded by his courtiers, his dignity the while suffering no disturbance. Few persons unpracticed in state craft would dare venture the experiment. Cæsar, booted and spurred, leading his victorious legions through Gaul, was a totally different man from the Cæsar who earned the contempt of Brutus by his puling conduct when he had the fever in Spain.

The recumbent position is one no more favorable to seeing objects correctly with the mental than the bodily vision. Who that has had occasion to put to paper the ideas that thrust themselves upon the mind during the waking hours of night, has not been disappointed at the show they make when subjected to such a test? This morbid coloring of ideas, thus engendered, may be in part owing to the influence of the silence and darkness of night, though it is enough perceived in the day time to show that it is largely due to mere recumbency of position. Even to the most vigorously constituted, the bed is the habitation of fears and apprehensions, which vanish when the subject of them faces the world in the attitude of encounter.

The terms applied by different individuals to the same degree of personal suffering vary to the widest extremes; and the physician should be carefully on his guard against those whose minds conceive such a state only in the superlative degree. With some, a pain is always "terrible;" "beyond all endurance;" or, it is "torturing," "racking," "excruciating," as if the inventions of martyrdom could only supply fitting terms for its expression, while another, in defining the same amount of suffering, uses only the proper positive. The difficulty in correct diagnosis, is increased where the individual makes his act, while under observation, correspond with his language. He turns himself in bed with a groan, starts at the examining touch, catches his breath at each inspiration, distorts his countenance, or walks with his hand pressed carefully to his side.

Perhaps a more frequent form of this deception is in regard to the exercise of some physical function. Some habitually attempt to deceive in regard to the excretions, their frequency and amount, using, sometimes, the most loathsome devices to conceal the real fact. More frequently it is in regard to the amount of nutriment taken. Misrepresentations in regard to the latter fact, are frequent, beyond the idea even of the most suspicious. Multitudes of weak-minded persons seem to regard abstinence from food as something meritorious, and deceive, with regard to it, for the sake of winning the sympathy of others. The same morbid disposition will often lead patients to falsify with regard to the operation of medicines. This is true, particularly of cathartics, and the prescriber is frequently led to increase the dose, even to an injurious amount.

Great skill is required to separate the fictitious from those real suspensions of physicial functions with which mental disease is so frequently connected. The suspension of physical pain, for instance, which occurs in many cases of mental disease, is so great as to mask and conceal bodily injuries of an extreme character. The nerves of sensation seem actually paralyzed. For many years the apparent fact seemed remarkable that very few insane persons died of phthisis pulmonalis. The disuse of autopsies in insane hospitals allowed that error long currency, till it was shown such by the investigations of a gentleman* connected with an institution where that practice was maintained. From his investigations it is shown that pulmonary disease exists quite as much among the insane as among others,—the cough, pains in the chest, &c., being absent merely through a blunted condition of the nerves of sensation. For the same reason, fractures and other serious injuriès received by insane persons, often escape detection till some time after they are received into institutions for the treatment of mental disease.

The history of cases of insanity presented for treatment often reminds us how frequently the disease has commenced with some delusion upon the subject of health; and the time spent in the treatment of a disease wholly imaginary, and the number of practitioners who will be successfully deceived by the same case, is matter of continual surprise. The forms which such imaginary diseases assume are truly Protean, and practitioners are much to blame for their readiness to give a name for the thousand shifting and transient sensations to which all persons of ill-regulated sensibilities are more or less subject.

The unthinking practitioner who gives a name to an array of sensations which may be detailed to him, merely for the sake of being rid of a troublesome consulter, and without hav-

* Dr. Joseph Workman, Toronto, Canada.

ing some grounds for such a diagnosis though a satisfying examination, often does an injury which no time can remedy. As an opinion in favor of a delusion will have more weight than many against it, he who, by a professional opinion, gives a local habitation and a name to what was before an airy nothing of the imagination, does a fellow-being a lasting, and may be, fatal injury.

An individual whose vagaries of sensation have thus become magnified into a disease, becomes one of the most miserable of all the mild class of lunatics, as much from the new name which his fancied disease is perpetually getting, or the new disease which each successive prescriber adds to the already appalling host, as from his actual sufferings. His disease is dyspepsia, heart disease, liver complaint, or marasmus, as the case may be, and thus the poor victim finds himself running down a page of nosological horrors, the tendency of which is by no means to lessen the speed by which he is hastening to the inevitable conclusion. For years he will be passing from one physician to another, till his faith in the faculty is exhausted; then through the various forms and grades of empiricism, till every function of his body is completely vitiated by such an unnatural experience. On reaching the lunatic asylum, which is the usual goal of such a course, we find, in the verdict of the jury which commits him, the same stereotyped cause standing forth, like the skull and crossbones of old time tombstones, to wit: "ill health."

Young and middle-aged men are, probably, most frequently the subjects of this mental disease, and no examination is complete which does not include a careful scrutiny as to the existence of vicious habits of a solitary nature, when such cases present themselves. And here let me caution as to the general unreliability of all statements which such subjects may make as it regards this conclusive fact. No exterior of respectability, no professions of better things, no previous character for veracity and candor seem proof against the spirit of mendacity which this detestable practice appears to create. It is only when the individual becomes thoroughly alarmed that the truth comes out of him.

Somewhat nearly allied to the last, though less frequently resulting in positive insanity, is that perpetually existing and utterly incurable malady, chiefly occurring among females, and affording to such practitioners as give it their encouragement, no small amount of their employment. It is a sort of medico-mania, an unquenchable desire to make of themselves a constant thoroughfare for drugs. Some real illness may have at first called the habit into existence, though it requires to bring the case to perfection, a constitutional predisposition to it. It is so frequently found prevalent in particular families as to support the idea that, like graver mental maladies, it is a matter of inheritance. With such persons the medical idea seems to fill the mind. The chronicles of neighboring sickness, past and present, and the sayings and doings of some favorite practitioner, are the principal topics of their conversation, with as full a narration of their own diseases added as the time and stomach of any listener can be found to bear. To get up about themselves the atmosphere of the sick-room seems to be their highest delight, and the pleasurable eras in their lives are when they have set on foot the liveliest anxieties, and produced the widest outflow of physic and sympathy. Their imbibition of medicine really never ceases, and, as the articles which they most affect are of the class of diffusible stimulants, they at last become as necessary as the dram of the inebriate. Their diseases are as far from any nosological distinction as those of the last named class. They are really in a great degree imaginary, and differ little from other mental infirmities.

In the class alluded to before the last, should also have been included cases that for a long time deceive, which show themselves by a disposition to go to bed, with little actual complaint of illness, and become fixed in the habit sometimes for long periods. Perhaps, it is more frequently an adhesion to the bed after some actual disease has run its course. This form may appear in males or females alike; perhaps, more frequently in the latter, and, when found in the former, generally, I apprehend, connected with indulgence in secret vice. So averse is the individual sometimes to being seen, that physicians, and even near neighbors, are unaware, perhaps for years, as I have known, that the missing person is not away from home. That this is a distinct form of insanity is proven by its occurrence in families, other members of which have shown insanity in other forms, and also by its frequently being found as the introduction to more manifest insanity.

The extent to which such persons will sometimes impose their imaginings upon others as realities, is one of the curiosities of human experience. The patient martyrdom of the sympathizing mother, regarding it as her pious duty to forego every earthly pleasure in order to confine herself to the bedside of a daughter thus afflicted, whose condition constantly becomes more deplorable by witnessing this very self-devotion on the part of the parent, is a truly affecting instance of a double delusion, in which it is difficult to say which case is the more pitiable.

We leave out of consideration the whole class of *quasi* mental disorders that show themselves in connection with hysteria, though they might form an interesting chapter in this paper, and pass to notice another peculiar malady of the mind that occurs as a sequel to the puerperal state.

Well-marked puerperal insanity is a disease peculiarly rife in this section of the country, to judge from an experience enabling a comparison with widely different localities. By far the most frequent of all the forms of this disease, is the chronic and hardly recognized one, which is found treated by no author, the distinguishing feature of which is a change in the disposition of the person affected, especially in whatever concerns the social relations, the domestic affections, and the moral tendencies. In the lighter form of this disease, those who observe it are perplexed at the phenomena which it presents. A lady, affected by this form of disease, is found to have suffered a remarkable change, dating from some previous confinement. Traits of character appear, hitherto unknown by those most in her intimacy. She becomes irritable, subject to causeless fits of passion, and jealous of, and estranged from those in whom she had before invested fullest confidence. Sometimes she is merely changed in temperament, and is moody, solitary, and reserved. These symptoms have their aggravation whenever the functions of the uterine system are in action, till a regular monthly fit of spleen, or something worse, becomes habitual. With neighbors and casual visitors, no change is visible, the power of self-control remaining till long after the disease is fully established, and often no interruption of domestic tranquillity is suspected till a sudden disruption takes place.

More rarely, this form of disease is exhibited in a change of disposition as it regards moral acts. Manifestations of dishonesty, disregard of truth, or moral impurity, stand in strange contrast with all the individual's antecedents. It is among the thousand forms of this peculiar disease that those cases appear which afford the most plausible instances of what has unfortunately been styled *moral insanity.*

And here, somewhat out of its natural order in this paper, a few observations may be proper upon the much vexed question, whether there be such a diseased condition of the human economy as fitly to be styled a moral insanity. It is not merely a speculative question, as those well understand who are much conversant with courts of justice.

In treating the associated insane, one is at once struck with the vast proportion of their aberrations which bear the aspect of mere moral perversity. A disposition wantonly inclined to create the greatest amount of trouble possible to others, an apparent delight in contemplating the mischief and destruction which their own hands have wrought, a seeming absence of even a vestige of the sentiments of gratitude, affection, or of the instinct of love as found even in the lower orders of animals; in short, a general hardness of the whole moral nature seems much more to distinguish a great number of cases than any disturbance in the power of memory, perception, or judgment, or of that class of faculties, which, in their entire state, constitute what is termed reason. Indeed, in very many instances, where these latter components of an entire mind have been restored, this seemingly diseased state of the moral sentiments will remain as if it constituted the very foundation of the abnormal phenomena of the case. I suspect, moreover, that the efficiency of the treatment obtained in an institution expressly for the insane, is much more shown in the removal of what may be styled the intellectual aberrations, than of the moral perversities of which we make mention. The constant observation of these facts has, probably, led some observers to conceive that a state might exist, deserving the name of an insanity, in which the mental operations, strictly considered, remained wholly unimpaired. The admission of such a state of facts is a matter of great magnitude. There could be no limit set to its conclusions short of an embrace of a large share of what we consider the catalogue of crime. Already some pretenders to psychological science have thrown reproach upon the entire plea of insanity in criminal cases, by substituting the captivating name of moral insanity for what is nothing else but sheer villany. No instance has fallen under my observation where any man of professional standing as a psychopathist has maintained this doctrine in any court of justice,—the only place where it assumes high practical importance. Yet, to apply the term "moral" to insanity in the general, or to any of its forms, is virtually conceding a great deal too much, unless we are willing to concede a great deal more. As a mere philological matter, the word "moral" may be applied to insanity as well as the word "mental;" it is the question whether the application had better not be made, where it is required, by the religious teacher, in whose province it lies. But *our* insanity,—the insanity of the psychopathist and the physician, the insanity as treated by the great authors on the subject, and for whose cure insane asylums are founded,—has a meaning which is in part revolutionized when the adjective "moral" is made its prefix.

It is an undecided question, whether what are called the moral characteristics have some distinct existence, and can be separately considered and treated of, or whether they are the fruit, so to speak, of certain mental processes. Nothing would be gained by an attempt to argue a point of such nicety, and where so little of a conclusion could ever be reached, and the better way will be to inquire whether a case answering our idea of the disease is ever seen. We cannot call anything moral insanity, except an impulse to do wrong or criminal acts, so uncontrollable by the processes of reason,—themselves being unimpaired,—as to amount to a disease. Any appreciable disturbance of mental integrity of course puts the case in another category.

To show how rare such a condition must be, I have carefully reviewed about twenty-four hundred cases of insanity treated, and am unable to recall a single case possessing even the general features of the ideal which the mind conceives as the disease in question. Dr. Workman, the Superintent of the Provincial Lunatic Asylum, Toronto, C. W., cites a case in the April number of the *American Journal of Insanity*, as being the first case of the kind found in two thousand cases treated. It is evident, in the narration of this case, that the author of the article describing it has some misgivings as to its nature; and a careful reading of its description will lead many others to the belief that Solomon's remedy for moral obliquities would have been, in this case, the suitable one. Now, here we have one case cited in an aggregate of forty-four hundred, and that a doubtful one. Is that a percentage worth basing a nosological distinction upon?

When we examine those cases which are cited by authors who treat of this disease, the conviction is forced upon us that in many, if not most of them, there is real intellectual disturbance, though masked by the stronger manifestation of moral perversity; and that these writers have fallen into the common and very natural error of making some isolated, though very impressive case, stand as the representative of an imagined class.

It has always seemed as if all that is included in the idea of moral insanity, might be better disposed of by a closer reference to some phenomena of insanity which are of every day experience, than by recognizing a distinct disease, the support of which involves so much of perplexity.

Every one realises how few of the delusions of the insane mind are ever revealed, and how readily they are revealed under one set of circumstances and concealed under others. All insane asylums abound in cases of unquestionable mental disease, where its palpable manifestations are so obscure that the unskilled observer would doubt its existence. A certain suspicious reserve, a mysterious shyness of manner, some haughtiness of bearing, or something marked and singular in tone of voice and manner of utterance, some strange attachment to some particular position or seat, or special stress applied to the doing of some act, may be all that distinguishes the individual from other men. Yet one guided by experience, has no hesitation in declaring such cases to be instances of a latent delusion, and is prepared for the sudden exhibition of extreme or violent acts, of which any of these almost unobserved antecedent peculiarities furnishes the explanatory key. In such cases, the extent of the disease is not at all measured by what appears on the surface, and those who treat the insane are constantly surprised by the revelations of recovered patients, as to the multitude and singularity of the delusions which possessed them while in a state which seemed, for all discoverable signs, so little removed from full enjoyment of reason. The delusion may be, indeed, completely latent, having no outward manifestation whatever, and yet may give rise to all those singular, inexplicable, and, perhaps, criminal acts, which a failure to explain by any accompanying indications of delusion has styled moral insanity. It is very easy to conceive a case possessing the declared attributes of the disease called in question; but before admitting the fact, the possibility of a latent delusion underlying its characteristic perversities of conduct, should be well considered.

It may be said, in reply to this view of the subject, that it assigns to delusion too indispensable a place in all cases of insanity, whereas it is well known that in a vast multitude of cases it has no demonstrable presence. This does not necessarily follow. Delusion among the insane may be supposed to bear about the same relative part in their unnatural acts that a well-defined motive does in the acts of those who reason correctly. Persons possessed of reason perform the larger portion of their acts from no well-considered motive of which they are conscious. Acts are done from an impulse which is, after all, the result of some former reasoning process. So the phenomena of moral insanity, so called, may follow some former diseased process of thought of which the individual himself has no consciousness, and which, of course, no skill of another could detect.

Another explanation of the phenomena termed moral insanity should not be lost sight of. We are apt to forget the vast conservative power of reason in saving man from the depraved appetites and instincts common to him with the brute creation. Swift has well shown the humiliation of our species, when man's reason was given to the brute and himself left without it. We all remember, in the entertaining narrative of Captain Gulliver, what a sorry brute man becomes when thus transformed. A human being, born without reason, or possessing it only to a low degree, becomes an instance such as we often see illustrating this point. The instincts of the idiot are low, and are prevented from becoming depraved only by the amount of reason which he has. The small degree of reason that he possesses may educate the faculties of fear, of censure, and punishment, and love of approbation, and may cause him to imitate his superiors by a propriety of conduct that may set him above criminal acts. The same power exerted over the moral propensities by the processes of pure reasoning, is also shown in the cases of children. Childhood, notwithstanding the praises bestowed on it as the unsullied spring-time of existence, does not compare with mature age in the rightfulness of its acts. The burglary and murder of birds-nesting peculiarly gratifies the juvenile heart, and how often must the ghost of the family cat, done to death by truant hands, haunt the little murderer's pillow. Whoever has looked, too, upon a quarrel in petticoats, waged for a bit of cake, sees a ferocity as great almost as the death-struggle of mortal foes. Yet, what but the power of pure reason, working through years, changes these robbers, murderers, falsifiers, and belligerents into discriminating judges, and reverend dispensers of the gospel of mercy and peace? And how easily and naturally will an inclination to those same acts return when that essence which has rescued from them is withdrawn.

Hence the position taken, that moral insanity, if by that term is meant a disease of the affective faculties, in which the intellect has no share, has no proved existence; and that what has received that appellation is nothing more than either the result of a latent, undetected delusion, whose *modus operandi* we are unable to demonstrate, or the passive effect of a weakened influence of the reasoning powers over man's baser instincts.

A review of minor mental maladies would be incomplete without mention of that form of well-known mental disease which will be more quickly recognized from some of its leading features than by any name that could possibly apply to it. It seems to consists in a love for the extreme, the eccentric, and the general opposite to the received opinions, practices, and fashions of the rest of mankind. One would believe this class to be much more numerous than it really is, from the facility with which a single individual, having played himself out in one character, turns up in another. To him, all the world is, literally, a stage, which he crosses every time the last new, strange idea finds a lodgment in his quickly receptive, but perfectly non-retentive brain. In religion, he follows a side track, with none but his kindred motley associates, no two of whom agree, except in the common opposition to everything established by the concurrence of the rest of mankind. In politics, he is so far advanced from every body else that he is rarely overtaken, and should he be, he disappears entirely, his occupation being gone the moment he finds the world at the same goal with himself. This class possesses affinities of its own; it has its own special literature,—a something, part medical, part religious, and part politico-economical,—and if not actually a species of insanity, it is the best recruiting ground for the insane asylum.

A solitary life is not only the surest preparation for mental disease of the most unpromising kind, but where it exists from confirmed choice may, of itself, be regarded as a species of insanity. No one is safe from the visitation of mental disease who allows one of the natural connections which hold him in his place, as a social being, to be broken. Decidedly the most powerful conservator of reason in the individual is that constant exercise of the moral and mental faculties which a close relation to society creates. Those whose habits or associations hold society at arm's length, must always be considered in peril. Happy is the man, in this point of view, whose daily bread comes from the hands of those with whom he daily associates. A true record would show that a large part of the eccentricities, irritabilities, and consequent calamities of authors, arise less from the close kindred of wit and madness than from the unlucky ability to draw fame and fortune from a distance.

If I were required to produce a lunatic to order, I would take, as the raw material, the college student in his bachelor hall, provided with his needle-book, spools, and the inevitable bag of buttons. Buttons, I grant, are good, but if they are simply holding together the lapels of coats, and are having no part in the social commerce between awkward dependence and quick and tender sympathy, they are as naught. Having thus established a social non-conductor, if I then could introduce into my subject some strange element of religious belief, some crotchet of unheard-of philosophy, or, even some *outre* taste in matters of every-day life, I could safely lay my work up to dry, fully confident that time would do the rest.

A recognition of these various forms of mental disease is of especial importance in their relation to medical jurisprudence. This department of medical science is shorn of much of its value in shaping the administration of justice, by overlooking the importance of these minor mental maladies in multiplying infractions of the law. The physician is always ready enough to throw the influence of his opinion into the scale where great crimes have attending circumstances, out of which science can show proof of irresponsibility from mental disease; but he is not so often willing to ask himself the question, in lesser offences, how much of mitigation there may be in some incipient mental malady which none suspect, and which it is no one's business to think of but his. To my own certain knowledge, the courts of this State are continually sending palpable insane men to its penitentiary, from which only their lack of profit as workers releases them. We cannot say that medical science has fully vindicated itself, till this blemish on our civilization is wiped away.

It was the remark of one, not more distinguished for his large acquaintance with the insane than for his eminence in other respects, that "an insane asylum is the best of all standing proofs of the doctrine of special providences." This remark implies a wonder at the small number of casualties that attend the association of many individuals, so large a proportion of whom are moved by the most dangerous promptings; and, among the surprises before alluded to, on hearing from convalescent patients of the strange hallucinations that have attended their disease, not the least is that they so unfrequently carry the delusions which possess them, into violent acts; and so far from being jealous of the introduction of the plea of insanity in criminal cases, our surprise is that it does not oftener occur.

It is one of the duties of medical men to keep themselves, in some degree, acquainted with their local court calendar of criminal trials, and be vigilant lest the heavy wheel of justice tread into the mire some broken but blameless spirit, whom no friendly hand is outstretched to save.

He who "spake as never man spake," uttered, as the most solemn of all his reproaches, "I was sick and in prison, and ye visited me not." As the insane are sick in a double sense, how deep the obligation becomes to every conscientious mind.

At this time it is the fashion to decry the medical expert who lifts up his voice from the witness-stand, to temper the stroke of justice as it descends on the head which God, in his mysterious wisdom, has already smitten. But sneering will no more blow away an eternal fact, established in a disease, than railing could lift the seal from the Jew's bond. The plea of insanity has found its firm stand at the criminal bar, and will continue to reäppear through all time; and the clear voice of medical science, heard above all the scoffers that time can engender, must be raised as long as it is true to the vocation with which it is called.

